# Supplementary material for: Heat adaptation of phage T7 under an extended genetic code
Source: Virus Evol. 2021 Dec 1;7(2):veab100. doi: 10.1093/ve/veab100 (PMC8923235; doi:10.1093/ve/veab100)
Supplement: veab100_Supp [file veab100_supp.zip › Sequencing_Analysis.zip!/Sequencing_Analysis/A1/evidence/SNP_10.html]

BRESEQ :: Evidence


| Predicted mutation | | | | | | |
| --- | --- | --- | --- | --- | --- | --- |
| evidence | seq id | position | mutation | annotation | gene | description |
| RA | NC\_001604.1 | 29,258 | A→G | intergenic (–/–) | – / – | –/– |

| Read alignment evidence... | | | | | | | | | | | |
| --- | --- | --- | --- | --- | --- | --- | --- | --- | --- | --- | --- |
|  | seq id | position | | ref | new | freq | score (cons/poly) | reads | annotation | genes | product |
| \* | NC\_001604.1 | 29,258 | 0 | A | G | 99.2% | 8545.9 / NA | 2787 | intergenic (–/–) | *–/–* | –/– |
| Reads supporting (aligned to +/- strand):  **ref** base A (5/4);  **major** base G (1632/1117);  **minor** base T (12/10);  **total** (1654/1133) | | | | | | | | | | | |
| Fisher's exact test for biased strand distribution *p*-value = 6.68e-01 | | | | | | | | | | | |
| Kolmogorov-Smirnov test that lower quality scores support variant *p*-value = 9.94e-01 | | | | | | | | | | | |

Only 100 of 2791 total aligned reads displayed.  

`GCGTCGTCCAGACTCTGCTGACTTCTTTGAGAAGTATATCGACAACGGTCTGGTTACTGGCGCAATCCCATCTGATGCTCAAGCCACACAGCTTATAAGCCAAGCGTTCAGTGACGCTTCTAGCCGTGCTGGTGGTGCTGACTTCCTGATGCGAGTCGGTGACAAGAAGGTAACACTTAACGGAGCCACTACGACTTACCGAGAGTTGATTGGTGAGGAACAGTGGAACGCTCTCATGGTCACAGCACAACGTTCTCAGTTTGAGACTGACGCGAAGCTGAACGAGCAGTATCGCTTGAAGATTAACTCTGCGCTGAACCAAGAGGACCCAAGGACAGCTTGGGAGATGCTTCAAGGTATCAAGGCTGAACTAGATAAGGTCCAACCTGATGAGCAGATGACACCACAACGTGAGTGGCTAATCTCCGCACAGGAACAAGTTCAGAATCAGATGAACGCATGGACGAAAGCTCAGGCCAAGGCTCTGGACGATTCCATGAAGTCAATGAACAAACTTGACGTAATCGACAAGCAATTCCAGAAGCGAATCAACGGTGAGTGGGTCTCAACGGATTTTAAGGATATGCCAGTCAACGAG  >  NC_001604.1/28960‑29557`  

                                                                                                                                                                                                                                                                                                          `|`                                                                                                                                                                                                                                                                                                             
 `cgtcgtCCAGACTCTGCTGACATCTTTGAGAAGTATATCGCCAACGGTCTGTGTAATGGCGCAATCCCATCTGAGGCTCAAGCCACACAGCTTATAAGCCAAGCGTTCAGTGACGCTTCTAGCCGTGCTGGTGGTGCTGACTTCCTGATGCGAGTCGGTGACAAGAAGGTAACCCTTAACGGAGCCACTACGACTTACCGAGAGTTGATTGGTGAGGAACAGTGGAACGCTCTCATGGTCACAGCACAACGTTCTCAGTTTGAGACTGACGCGAAGCTGAACGAGCAGTATCGCTTGgaga                                                                                                                                                                                                                                                                                                          <  1:94683/301‑1 (MQ=255)`  
  `gtcgtcCAGACTCTGCTGACTTCTTTGAGAAGTATATCGACAACGGTCTGGTTACTGGCGCAATCCCATCTGATGCTCAAGCCACACAGCTTATAAGCCAAGCGTTCAGTGACGCTTCTAGCCGTGCTGGTGGTGCTGACTTCCTGATGCGAGTCGGTGACAAGAAGGTAACACTTAACGGAGCCACTACGACTTACCGAGAGTTGATTGGTGAGGAACAGTGGAACGCTCTCATGGTCACAGCACAACGTTCTCAGTTTGAGACTGACGCGAAGCTGAACGAGCAGTATCGCTTGGAGAt                                                                                                                                                                                                                                                                                                         >  1:66353/1‑301 (MQ=255)`  
   `aagtcCAGACTCTGCTGACTTCTTTGAGAAGTATATCGACAACGGTCTGGTTACTGGCGCAATCCCATCTGATGCTCAAGCCACACAGCTTATAAGCCAAGCGTTCAGTGACGCTTCTAGCCGTGCTGGTGGTGCTGACTTCCTGATGCGAGTCGGTGACAAGAAGGTAACACTTAACGGAGCCACTACGACTTACCGAGAGTTGATTGGTGAGGAACAGTGGAACGCTCTCATGGTCACAGCACAACGTTCTCAGTTTGAGACTGACGCGAAGCTGAACGAGCAGTATCGCTTGGAGAtt                                                                                                                                                                                                                                                                                                        >  1:214690/3‑301 (MQ=255)`  
     `gtcCAGACTCTGCTGACTTCTTTGAGAAGTATATCGACAACGGTCTGGTTACTGGCGCAATCCCATCTGATGCTCAAGCCACACAGCTTATAAGCCAAGCGTTCAGTGACGCTTCTAGCCGTGCTGGTGGTGCTGACTTCCTGATGCGAGTCGGTGACAAGAAGGTAACACTTAACGGAGCCACTACGACTTACCGAGAGTTGATTGGTTAGGAACAGTGGAACGCTCTCATGGTCACAGCACAACGTTCTCAGTTTGAGACTGACGCGAAGCTGAACGAGCAGTATCGCTTGGAGATTaa                                                                                                                                                                                                                                                                                                      >  1:22679/1‑301 (MQ=255)`  
      `tcCAGACTCTACTGACTTCTTTGGGAAGTATAGCGACAACGGTCTGGTTACTGGCGCAAACCCATCTGATGCTCAAGCCACACAGCTTATAAGCCAAGCGTTCAGTGACGCTTCTAGCAGTGCTGGTGGTGCTGACTTCCTGATGCGAGTCGGTGACAAGAAGGTAACACTTAACGGAGCCACTACGACTTACCGAGAGTTGATTGGTGAGGAACAGTGGAACGCTCTCATGGTCACAGCACAACGTTCTCAGTTTGAGACTGACGCGAAGCTGAACGAGCAGTATCGCTTGGAGATTAAc                                                                                                                                                                                                                                                                                                     <  1:411336/301‑1 (MQ=255)`  
        `cAGACTCTGCTGACTTCGTTAAGAAATATATCGACAACTGTCTGGTTACTAGCGCAATCCCGTCTGATGCTCAAGCCACACAGATTATAAGCCAAGCGTTCAGTGACGCGTCTAGCCGTGCTGGTGGTGCTGACTTCCTGATGCGAGTCGGTGACAAGAAGGTAACACTTAACGGAGCCACTACGACTTACCGAGAGTTGATTTGTGAGGAACAGTGGAACGCTCTCATGGTCACAGCACAACGTTCTCAGTTTGAGACTGACGCGAAGCTGAACGAGCAGTATCGCTTGGAGATTAActc                                                                                                                                                                                                                                                                                                   <  1:352190/301‑1 (MQ=255)`  
             `gagatgtgtataagagacaggTATATCGACAACGGTCTGGTTACTGGCGCAATCCCATCTGATGCTCAAGCCACACAGCTTATAAGCCAAGCGTTCAGTGACGCTTCTAGCCGTGCTGGTGGTGCTGACTTCCTGATGCGAGTCGGTGACAAGAAGGTAACACTTAACGGAGCCACTACGACTTACCGAGAGTTGATTGGTGAGGAACAGTGGAACGCTCTCATGGTCACAGCACAACGTTCTCAGTTTGAGACTGACGCGAAGCTGAACGAGCAGTATCGCTTGGAGATTAACTCTgcgc                                                                                                                                                                                                                                                                                              <  1:152211/281‑1 (MQ=255)`  
              `ctgctgACTTCTTTGAGAAGTATATCGACAACGGTCTGGTTACTGGCGCAATCCCATCTGATGCTCAAGCCACACAGCTTATAAGCCAAGCGTTCAGTGACGCTTCTAGCCGTGCTGGTGGTGCTGACTTCCTGATGCGAGTCGGTGACAAGAAGGTAACACTTAACGGAGCCACTACGACTTACCGAGAGTTGATTGGTGAGGAACAGTGGAACGCTCTCATGGTCACAGCACAACGTTCTCAGTTTGAGACTGACGCGAAGCTTAACGAGCAGTATCGCTTGGAGATTAACTCTGCGCt                                                                                                                                                                                                                                                                                             >  1:74799/1‑301 (MQ=255)`  
               `agagacagtcttTGAGAAGTATATCGACAACGGTCTGGTTACTGGCGCAATCCCATCTGATGCTCAAGCCACACAGCTTATAAGCCAAGCGTTCAGTGACGCTTCTAGCCGTGCTGGTGGTGCTGACTTCCTGATGCGAGTCGGTGACAAGAAGGTAACACTTAACGGAGCCACTACGACTTACCGAGAGTTGATTGGTGAGGAACAGTGGAACGCTCTCATGGTCACAGCACAACGTTCTCAGTTTGAGACTGACGCGAAGCTGAACGAGCAGTATCGCTTGGAGATTAACTCTGCGCTg                                                                                                                                                                                                                                                                                            <  1:32461/293‑1 (MQ=255)`  
                     `cttcttTGAGAAGTATATCGACAACGGTCTGGTTACTGGCGCAATCCCATCTGATGCTCAAGCCACACAGCTTATAAGCCAAGCGTTCAGTGACGCTTCTAGCCGTGCTGGTGGTGCTGACTTCCTGATGCGAGTCGGTGACAAGAAGGTAACACATAACGGAGCCACTACGACTTACCGAGAGTTGATTGGTGAGGAACAGTGGAACGCTCTCATGGTCACAGCACAACGTTCTCAGTTTGAGACTGACGCGAAGCTGAACGAGCAGTATCGCTTGGAGATTAACTCTGCGCTGAACCaa                                                                                                                                                                                                                                                                                      >  1:133313/1‑301 (MQ=255)`  
                        `cttTGAGAAGTATATCGACAACGGTCTGGTTACTGGCGCAATCCCATCTGATGCTCAAGCCACACAGCTTATAAGCCAAGCGTTCAGTTACGCTTCTAGCCGTGCTGGTGGTGCTGACTTCCTGATGCGAGTCGGTGACAAGAAGGTAACACTTAACGGAGCCACTACGACTTACCGAGAGTTGATTGGTGAGGAACAGTGGAACGCTCTCATTGTCACAGCACAACGTTCTCAGTTTGAGACTGCCGCGAAGCTGAACGATCAGTATCGCTTGGAGATTAACTCTGCGCTGAACCAatag                                                                                                                                                                                                                                                                                   >  1:192627/1‑301 (MQ=255)`  
                        `cttTGAGAAGTATATCGACAACGGTCGGGTTACTGGCGCAATCCCATCTGATGCTCAAGCCACACAGCTTATAAGCCAAGCGTTCAGTGACGCTTCTAGCCGTGCTGGTGGTGCTGACTTCCTGATGCGAGTCGGTGACAAGAAGGTAACACTTAACGGAGCCACTACGACTTACCGAGAGTTGATTGGTGAGGAACAGTGGAACGCTCTCATGGTCACAGCACAACGTTCTCAGTTTGAGACTGACGCGAAGCTGAACGAGCAGTATCGCTTGGAGATTAACTCTGCGCTGAACCAagag                                                                                                                                                                                                                                                                                   <  1:333957/301‑1 (MQ=255)`  
                          `ttGAGAAGTATATCGGCAACGGTCTGGTTACTGGCGCAATCCCATCTGATGCTCAAGCCACACAGCTTATGAACCAAGCGTTCAATTACGCTTCTAGCCGTGCTGGTGGTGCTGACTTCCTGATGCGAGTCGGGGGAAAGAAGGTAACACTTAACGGAGCCACTACGACTTACAGAGAGTTGATTGGTGAGGAACAGTGGAACGCTCTCATTGTCACAGCACAACGTTCTCAGTTTGAGACTGACGCGAAGCTGAACGAGCAGTATCGCTTGGAGATTAACTCTGCGCTGAACCAAGAGGa                                                                                                                                                                                                                                                                                 <  1:220145/301‑1 (MQ=255)`  
                                 `gTATATCGACAACGGTCTGGTTACTGGCGCAATCCCATCTGATGCTCAAGCCACACAGCTTATAAGCCAAGCGTTCAGTGACGCTTCTAGCCGTGCTGGTGGTGCTGACTTCCTGATGCGAGTCGGTGACAAGAAGGTAACACTTAACGGAGCCACTACGACTTACCGAGAGTTGATTGGTGAGGAACAGTGGAACGCTCTCATGGTCACAGCACAACGTTCTCAGTTTGAGACTGACGCGAAGCTGAACGAGCAGTATCGCTTGGAGATTAACTCTGCGCTGAAACAAGAGGACCCCAgg                                                                                                                                                                                                                                                                          >  1:503759/1‑301 (MQ=255)`  
                                   `atatCGACAACGGTCTGGTTACTGGCGCAATCCCATCTGATGCTCAAGCCACACAGCTTATAAGCCAAGCGTTCAGTGACGCTTCTAGCCGTGCTGGTGGTGCTGACTTCCTGATGCGAGTCGGTGACAAGAAGGTAACACTTAACGGAGCCACTACGACTTACCGAGAGTTGATTGGTGAGGAACAGTGGAACGCTCTCATGGTCACAGCACAACGTTCTCAGTTTGAGACTGACGCGAAGCTGAACGAGCAGTATCGCTTGGAGATTAACTCTGCGCTGAACCAAGAGGACCCAAGGAc                                                                                                                                                                                                                                                                        <  1:161063/301‑1 (MQ=255)`  
                                   `atagCGACAACGGTCTGGTTACTGGCGAAATCCCAGCTGATGCTCAAGCCACACAGGTTATAAGCCAATGGGTCAGTGACGCTTCTAGCCGTGCTGTTGGTGCTGACTTACTGATGCGAGTCGGTGACAAGAAGGTAACACTTAACGGAGCCACTACGACTTACCGAGAGGTGATTGGTGAGGAACAGTGGAACGCTCTCATGGTCACAGCACAACGTTCTCAGTTTGAGACTGACGCGAAGCTGAACGAGCAGTATCGCTTGGAGATTAACTCTGCGCTGAACCAAGAGGACACAAGGAc                                                                                                                                                                                                                                                                        <  1:378701/301‑1 (MQ=255)`  
                                     `atCGACAACGGTCTGGTTACTGGCGCAATCCCATCTGATGCTCAAGCCACACAGCTTATAAGCCAAGCGTTCAGTGACGCTTCTAGCCGTGCTGGTGGTGCTTACTTCCTGATGCGAGTCGGTGACAAGAAGGTAACACTTAACGGAGCCACTACGACTTACCGAGAGTTGATTGGTGAGGAACAGTGGAACGCTCTCATGGTCACAGCACAACGTTCTCAGTTTGAGACTGACGCGAAGCTGACCGAGCAGTATCGCTTGGAGATTAACTCTGCGCTGAACCAAGCGGACCAAAGGACAg                                                                                                                                                                                                                                                                      >  1:103851/1‑301 (MQ=255)`  
                                      `agagacagggTCTGGTTACTGGCGCAATCCCATCTGATGCTCAGGCCACACAGCTTATAAGCCAAGCGTTCAGTGACGCTTCTAGCCGTGCTGGTGGTGCTGACTTCCTGATGCGAGTCGGTGACAAGAAGGTAACACTTAACGGAGCCACTACGACTTACCGAGAGTTGATTGGTGAGGAACAGTGGAACGCTCTCATGGTCACAGCACAACGTTCTCAGTTTGAGACTGACGCGAAGCTGAACGAGCAGTATCGCTTGGAGATTAACTCTGCGCTGAACCAAGAGGACCCAAGGACAGc                                                                                                                                                                                                                                                                     <  1:423399/293‑1 (MQ=255)`  
                                         `aCAACGGTCTGGTTACTGGCGCAATCCCTTCTGATGCTCAAGCCACACAGCTTATAAGCCAAGCGTTCAGTGACGCTTCTAGCCGTGCTGGTGGTGCTGACTTCCTGATGCGAGTCGGTGACAAGAAGGTAACACTTAACGGAGCCACTACGACTTACCGAGAGTTGATTGGTGAGGAACAGTGGAACGCTCTCATGGTCACAGCACAACGTTCTCAGTTTGAGACTGACGCGAAGCTGAACGAGCAGTATCGCTTGGAGATTAACTCTGCGCTGAACCAAGAGGACCCAAGGACAGCTTg                                                                                                                                                                                                                                                                  >  1:328356/1‑301 (MQ=255)`  
                                              `ggTCTTGTTACTGGCGCAATCCCATCTGATGCTCAAGCCACACAGCTTATAAGCCAAGCGTTCAGTGACGCTTCTAGCCGTGCTGGTGGTGCTGACTTCCTGATGCGAGTAGGTGACAAGAAGGTAACACTTAACGGAGCCACTACGACTTACCGAGAGTTGATTTGTGAGGAACAGTGGAACGCTCTCATGGTCACAGCACAACGTTCTCAGTTTGAGACTGACGCGAAGCTGAACGAGCAGTATCGCTTGGAGATTAACTCAGCACTGAACCAAGAGGACCCAAGGACAGCTTGGgaga                                                                                                                                                                                                                                                             <  1:143053/301‑1 (MQ=255)`  
                                                    `gTTACTGGCGCAATCCCATCTGATGCTCAAGCCACACAGCTTATAAGCCAAGCGTTCAGTGACGCTTCTAGCCGTGCTGGTGGTGCTGACTTCCTGATGCGAGTCGGTGACAAGAAGGTAACACTTAACGGAGCCACTACGACTTACCGAGAGATGATTGGTGAGGAACAGTGGAACGCTCTCATGGTCACAGCACAACGTTCTCAGTTTGAGACTGACGCGAAGCTGAACGAGCAGTATCGCTTGGAGATTAACTCTGCGCTGAACGAAGAGGACCCAAGGACAGCTTGGGAGATGCttt                                                                                                                                                                                                                                                       >  1:357780/1‑300 (MQ=255)`  
                                                        `cTGGCGCAATCCCATCTGATGCTCAAGCCACACAGCTTATAAGCCAAGCGTTCAGTGACGCTTCTAGCCGTGCTGGTGGTGCTGACTTCCTGATGCGAGTCGGTGACAAGAAGGTAACACTTAACGGAGCCACTACGACTTACCGAGAGTTGATTGGTGAGGAACAGTGGAACGCTCTCATGGTCACAGCACAACGTTCTCAGTTTGAGACTGACGCGAAGCTGAACGAGCAGTATCGCTTGGAGATTAACTCTGCGCTGAACCAAGAGGACCCAAGGACAGCTTTGGAGATGCTTCAAgg                                                                                                                                                                                                                                                   <  1:171536/301‑1 (MQ=255)`  
                                                        `cTGGCGCAATACCATCTGATGCTCAAGCCACACAGCTTATAAGCCAAGCGTTCAGTGACGCTTCTAGCCGTGCTGGTGGTGCTGACTTCCTGATGCGAGTCGGTGACAAGAAGGTAACACTTAACGGAGCCACTACGACTTACCGAGAGTTGATTGGTGAGGAACAGTGGAACGCTCTCATGGTCACAGCACAACGTTCTCAGTTTGAGACTGACGCGAAGCTGAACGAGCAGTATCGCTTGGAGATTAACTCTGCGCTGAACCAAGAGGACCCAAGGACAGCTTGGGAGATGCTTCAAgg                                                                                                                                                                                                                                                   <  1:30987/301‑1 (MQ=255)`  
                                                         `aggCGCAATCCCATCTGATGCTCAAGCCACACAGCTTATAAGCCAAGCGTTCAGTGACGCTTCTAGCCGTGCTGGTGGTGCTGACTTCCTGATGCGAGTCGGTGACAAGAAGGTAACACTTAACGGGGCCACTACGACTTACCGAGAGTTGATTGGTGAGGAACAGTGGAACGCTCTCATGGTCACAGCACAACGTTCTCAGTTTGAGACTGACGCGAAGCTGAACGAGCAGTATCGCTTGGAGATTAACTCTGCGCTGAACCAAGAGGACCCAAGGACAGCTTGGGAGATGCTTCAAggg                                                                                                                                                                                                                                                  <  1:248142/300‑2 (MQ=255)`  
                                                          `ggCGCAATCCCATCTGATGCTCAAGCCACACAGCTTATAAGCCAAGCGTTCAGTGACGCTTCTAGCCGTGCTGGTGGTGCTGACTTCCTGATGCGAGTCGGTGACAAGAAGGTAACACTTAACTGAGCCACTACGACTTACCGAGAGTTGATTGGTGAGGAACAGTGGAACGCTCTCATGGTCACAGCACAACGTTCTCAGTTTGAGACTGACGCGAAGCTGAACGCGCAGTATCGCTTGGAGATTAACTCTGCGCTGTACCAAGAGGACCCAAGGACAGCTTGGAAGATTCTTCAAGGTa                                                                                                                                                                                                                                                 >  1:56353/1‑301 (MQ=255)`  
                                                          `ggCGCAATCCCATCTGATGCTCAAGCCACACAGCTTATAAGCCAAGCGTTCAGTAACGCTTCTAGCCGTGCTGGTGGTGCTGACTTCCTGATGCGAGTCGGTGACAAGAAGGTAACACTTAATGGAGCCACTACTACTTACCGAGAGTTGATTGGTGAGGAACAGTGGAACGCTCTCATGGTCACAGCACAACGTTCTCAGTTTGAGACTGACGCGAAGCTGAACGAGCAGTATCGCTTGGAGATTAACTCTGCGCTGAACCAAGAGGACCCAAGGACAGCTTGGGAGATGCTTCAAGGTa                                                                                                                                                                                                                                                 >  1:119869/1‑301 (MQ=255)`  
                                                             `ggaagcccATCTAAGGCTCAGTCCACCAAGCTGATAAGCCAAGCGTTAAGTGACGCTTCTAGCCGTGCTAGGGTTGCTTACTTCCTGATGCGAGTCGGTGACAAGAAGGTAACACTTAGCGGATCCACTACGACTTACCGGGAGTTGATTGTTGAGGAAAAGTGGAACGCTCTAATGGTCACAGCACAACGTTCTCAGTTTGAGACTGACGCGAAGCGGAACGAGCAGTATCGCTTGGAGATTAACTCTGCGCTGAACCAAGAGGACCCAAGGACAGCTTGGGAGATGCTTCAAGGTATCa                                                                                                                                                                                                                                              <  1:285188/296‑1 (MQ=255)`  
                                                                `aTCCCATCTTATGCTCAAGCCACACAGCTTATAAGCCAAGCGTTCAGTGACGCTTCTAGCCGTGCTGGTGGTGCTGACTTCCTGATGCGAGTCGGTGACAAGTAGGTAACACTTAACGGAGCCACTACGACTTACCGAGAGTTGATTGGTGAGGAACAGTGGAATGCTCTCATGGTCACAGCACAACGTTCTCAGTTTGTGACTGACGCGAAGCTGAACGAGAAGTATCGCTTGGATATTAACTCTGCGCTGAACCAAGAGGACCCAAGGACAGCTTGGGAGATGCTTCAAGGTATCAAgg                                                                                                                                                                                                                                           >  1:43249/1‑301 (MQ=255)`  
                                                                `aTCCCATCTGATGCTCAAGCCACACAGCTTATAAGCCAAGCGTTCAGTGACGCTTCTAGCCGTGCTGGTGGTGCTGACTTCCTGATGCGAGTCGGTGACAAGAAGGTAACACTTAACGGAGCCACTACGACTTACCGAGAGTTGATTGGTGAGGAACAGTGGAACGCTCTCATGGTCACAGCACAACGTTCTCAGTTTGAGACTGACGCGAAGCTGAACGAGCAGTATCGCTTGGAGATTAACTCTGCGCTTAACCAAGAGGACCCAAGGACAGCTTGGGAGATGCTTCAAggctgtctct                                                                                                                                                                                                                                           >  1:396516/1‑293 (MQ=255)`  
                                                                 `tcacATCTGATGCTCAAGCCACACAGCTTATAAGCCAAGCGTTCAGTGACGCTTCTAGCCGTGCTGGTGGTGCTGACTTCCTGATGCGAGTCGGTGACAAGAAGGTAACACTTAACGGAGCCACTACGACTTACCGAGAGTTGATTGGTGAGGAACAGTGGAACGCTCTCATGGTCACAGCACAACGTTCTCAGTTTGAGACTGACGCGAAGCTGAACGAGCAGTATCGCTTGGAGATTAACTCTGCGCTGAACCAAGAGGACCCAAGGACAGCTTGGGAGATGCTTCAAGGTATCAAGGc                                                                                                                                                                                                                                          <  1:70855/298‑1 (MQ=255)`  
                                                                 `gggctcggagatgtgtataagagacagttATAAGCCAAGCGTTCAGTGACGCTTCTAGCCGTGCTGGTGGTGCTGACTTCCTGATGCGAGTCGGTGACAAGAAGGTAACACTTAACGGAGCCACTACGACTTACCGAGAGTTGATTGGTGAGGAACAGTGGAACGCTCTCATGGTCACAGCACAACGTTCTCAGTTTGAGACTGACGCGAAGCTGAACGAGCAGTATCGCTTGGAGATTAACTCTGCGCTGAACCAAGAGGACCCAAGGACAGCTTGGGAGATGCTTCAAGGTATCAAGGc                                                                                                                                                                                                                                          <  1:406240/274‑1 (MQ=255)`  
                                                                 `ccccATCTGCTGCTCAAGCCACCCAGCGTATAAGCCAAGCGTTCAGTGACGCTTCTAGCCGTGCTGGTGGTGCTGACTTCCTGATTCGAGGCGGGGACAAGAAGGTAACACTTAACGGAGCCACTACGACTTACCGATAGTTGATTGGTGAGGAACAGTGGAACGCTCTCATGTTCACAGCACAACTTTCTCAGTTTGAGACTGACGCGAAGCTGAACGAGCAGTATCGCTTGGAGATTAACTCTGCGCTGCACCAAGAGGACCCAAGTACAGCTTGGGAGATGCTTCAAGGTATCAAGGc                                                                                                                                                                                                                                          <  1:186022/300‑1 (MQ=255)`  
                                                                 `acagaccTGATGCTCAAGCCACACAGCTTATAAGCCAAGCGTTCAGTGACGCTTCTAGCCGTGCTGGTGGTGCTGACTTCCTGATGCGAGTCGGTGACAAGAAGGTAACACTTAACGGAGCCACTACGACTTACCGAGAGTTGATTGGTGAGGAACAGTGGAACGCTCTCATGGTCACAGCACAACGTTCTCAGTTTGAGACTGACGCGAAGCTGAACGAGCAGTATCGCTTGGAGATTAACTCTGCGCTGAACCAAGAGGACCCAAGGACAGCTTTGGAGATGCTTCAAGGTATCAAGGc                                                                                                                                                                                                                                          <  1:401277/295‑1 (MQ=255)`  
                                                                  `cccATCTGATGCTCAAGCCACACAGCTTATAAGCCAAGCGTTCAGTGACGCTTCTAGCCGTGCTGGTGGTGCTGACTTCCTGATGCGAGTCGGTGACAAGAAGGTAACACTTAACGGAGCCACTACGACTTACCGAGAGCTGATTGGTGAGGAACAGTGGAACGCTATCATGGTCACAGCACAACGTTCTCAGTTTGAGACTGACGCGAAGCTGAACGAGCAGTATTGCTTGGAGATTAACCCTGCGCTTAACCAAGAGCACCCAAGGACAGCCTGGGGGATGCTTCAAGGTATCAAGGCt                                                                                                                                                                                                                                         >  1:45847/1‑301 (MQ=255)`  
                                                                     `aTCTGATGCTCAAGCCACACAGCTTATAAGCCAAGCGTTCAGTGACGCTTCTAGCCGTGCTGGTGGTGCTGACTTCCTGATGCGAGTCGGTGACAAGAAGGTAACACTTAACGGAGCCACTACGACTTACCGAGAGTTGATTGGTGAGGAACAGTGGAACGCTCTCATGGTCACAGCACAACGTTCTCAGTTTGAGACTGACGCGAAGCTGAACGAGCAGTATCGCTTGGAGATTAACTCTGCGCTGAACCAAGAGGACCCAAGGACAGCTTGGGAGATGCTTCAAGGTATCAAGGCTGaa                                                                                                                                                                                                                                      >  1:167387/1‑301 (MQ=255)`  
                                                                            `gCTCAAGCCACACAGCTTATAAGCCAAGCGTTCAGTGACGCTTCTAGCCGTGCTGGTGGTGCTGACTTCCTGATGCGAGTCGGTGACAAGAAGGTAACACTTAACGGAGCCACTACGACTTACCGAAAGTTGATTGGTGAGGAACAGTGGAACGCTCTCATGGTCACAGCACAACGTTCTCAGTTTGAGACTGACGCGAAGCTGAACGAGCAGTATCGCTTGGAGATTAACTTTGCGCTGAACCAAGAGGACCCAAGGACAGCTTGGGAGATGCTTCAAGGTATCAAGGCTGAAcctgtct                                                                                                                                                                                                                               >  1:49479/1‑295 (MQ=255)`  
                                                                                      `cagaagttAAAAGCCAAGCGTTAAGTGACGCTTCTAACCGTGCTGGGGGTGCTGACTTCATGATGCGAGTAGGTGACAAGAAGGTAACACTGAACGGAGCAAAGACGACTTAACGAGAGTTGATTGGTGAGGAACAGTGGAACGCTCTCATGGTCACAGCACAACGTTATCAGGTTGAGACTGACGCGAAGCTGAACGAGCAGTATCGCTGGGAGATTAACTCTGCGCTGAACCAAGAGGACCCAAGGACAGCTTGGGAGATGCTTCAAGGTATCAAGGCTGAACTAGATAAGGTCCAAcc                                                                                                                                                                                                                     <  1:46385/295‑1 (MQ=255)`  
                                                                                      `aaaaGCTTATAAGCCAAGCGTTCAGTGACGCTTCTAGCCGTGCTGGTGGTGCTGACTTCCTGATGCGAGGCGGTGACAAGAAGGTAACACTTAACGGAGCCACTACGACTTACCGAGAGTTGATTGGTGAGGAACAGTGGAACGCTATCATGGTCACAGCACAACGTTCTCAGTTTGAGACTGACGCGAAGCTGAACGAGCAGTATCGCTTGGAGATTAACTCTGCGCTGAACCAAGAGGACCCAAGGACAGCTTGGGAGATGCTTCAAGGTATCAAGGCTGAACTAGATAAGGTCCAAcc                                                                                                                                                                                                                     <  1:274941/298‑1 (MQ=255)`  
                                                                                        `caGCTTATAAGCCAAGCGTTCAGTGACGCTTCTAGCCGTGCTGGTGGTGCTGACTTCCTGATGCGAGTCGGTGACAAGAAGGTAACACTTAACGGAGCCACTACGACTTACCGAGAGTTGATTGGTGAGGAACAGTGGAACGCTCTCATGGTCACAGCACAACGTTCTCAGTTTGAGACTGACGCGAAGCTGAACGAGCAGTATCGCTTGGCGATTAACTCTGCGCTGAACCAAGAGGACCCAAGGACAGCTTGGGAGATGCTTCAAGGTATCCAGGCTGAACTAGATAAGGTCCAACCtg                                                                                                                                                                                                                   >  1:115394/1‑301 (MQ=255)`  
                                                                                          `gCTTATAAGCCAAGCGTTCAGTGACGCTTCTAGCCGTGATGGTGGTGCTGACTTCCTGATGCGAGTCGGTGACAAGAAGGTAACACTTAACGGAGCCACTACGACTTACCGAGAGATGATTGGTGCGGAACAGTGGAACGCTCTCATGGTCACAGCACAACGTTCTCAGTTTGAGACTGACGCGAAGCTGAACGAGCAGTATAGCTTGGAGATTAACTCTGCGCTGAACCAAGAGGACCCAAGAACAGCTTGGGAGATGCTTCAAGGTATCAAGGCTGAACTCGATAAGGTCAACCCtgat                                                                                                                                                                                                                 >  1:90829/1‑301 (MQ=255)`  
                                                                                           `cgtataAGCCAAGCGTTCAGTGACGCTTCTAGCCGTGCTGGTGGTGCTGACTTCCTGATGCGAGTCGGTGACAAGAAGGTAACACTTAACGGAGCCACTACGACTTACCGAGAGTTGATTGGTGAGGAACAGTGGAACGCTCTCATGGTCACAGCACAACGTTCTCAGTTTGAGACTGACGCGAAGCTGAACGAGCAGTATCGCTTGGAGATTAACTATGCGCTGAACCAAGAGGACCCAAGGACAGCTTGGGAGATGCTTCAAGGTATCAAGGCTGAACTAGATAAGGTCCAACCtgatg                                                                                                                                                                                                                <  1:146108/299‑1 (MQ=255)`  
                                                                                              `ccaggCCAAGCGTTCAGTGACGCTTCTAGCCGTGCTGGTGGTGCTGACTGCCTGATGCGAGTCGGTGACAAGAAGGTAACACTTAACGGAGCCACTACGACTTACCGAGAGTTGACTGGTGAGGAACAGTGGAACGCTCTCATGGTCACAGTACAACGTTCTCAGTTTGAGACTGACGCGAAGCTGAACGAGCAGTATCGCTTGGAGATTAACTCTGCGCTGAACCAAGAGGACCCAAGGACAGCTTGGGAGATGCTTCAAGGTATAAAGGCTGAACTAGATAAGGTCCAACCTGATGAGc                                                                                                                                                                                                             <  1:416377/297‑1 (MQ=255)`  
                                                                                                 `aGCCAAGCGTTCAGTGACGCTTCTAGCCGTGCTGGTGGTGCTGACTTCCTGATGCGAGTCGGTGACAAGAAGGTAACACTTAACGGAGCCACTACGACTTACCGAGAGTTGATTGGTGAGGAACAGTGGAACGCTCTCATGGTCACAGCACAACGTTCTCAGTTTGAGACTGACGCGAAGCTGAACGAGCAGTATCGCTTGGAGATTAACTCTGCGCTGAACCAAGAGGACCCAAGGACAGCTTGGGAGATGCTTCAAGGTATCAAGGCTGAACTAGATAAGGTCCAACCTGATGAGCAGa                                                                                                                                                                                                          >  1:445462/1‑301 (MQ=255)`  
                                                                                                   `ccAAGCGTTCCGTGACGCTTCGAGCCGTGGTGGTGGTGCTGACTGCCTGATGCGAGTCGGTGACAAGAATGTAACACTTAACGGAGCCACTACGACTTACCGAGAGTTGATTGGTGAGGAACAGTGGAACGCTCTCATGGTCACAGCACAACGTTCTCAGTTTGAGACTGACGCGAAGCTGAACGAGCAGTATCGCTTGGAGATTAGCTCTGCGCTGAACCAAGAGGACCCAAGGACAGCTTGGGAGATGCTTCAAGGTATCAAGGCTGAACTAGATAAGGTCCAACCTGATGAGCAGATg                                                                                                                                                                                                        <  1:129796/301‑1 (MQ=255)`  
                                                                                                     `agaagtgtataagagacagtAGCCGGGCGGGGGGTGCTGACTTGCTGAGGCGGGACGGTGAAAAGAAGGTAACACTTAACGGAGCAACTACGACTTAGCGAGAGTTGATTGGTGAGGAACAGTGGAACGGTCTCATGGTCACAGCACAACGTTCTCAGTTTGAGACTGACGCGAAGCTGAACGAGCAGTATCGCTTGGAGATTAACTCTGCGCTGAACCAAGAGGACCCAAGGACAGCTTGGGAGATGCTTCAAGGTATCAAGGCTGAACTAGATAAGGTCCAACCTGATGAGCAGATGac                                                                                                                                                                                                      <  1:51212/282‑1 (MQ=255)`  
                                                                                                     `aaGCGTTCAGTGACGCTTCTAGCCGTGCTGGTGGTGCTGACTTCCTGATGCGAGTCGGTGACAAGAAGGTAACACTTAACGGAGCCACTACGACTTACCGATAGTTGATTGGTGAGGAACAGTGGAACGCTCTCATGGTCACAGCACAACGTTCTCAGTTTGAGACTGACGCGAAGCTGAACGAGCAGTATCGCTTGGAGATTAACTCTGCGCTGAACCAAGAGGACCCAAGGACAGCTTGGGAGATGCTTCAAGGTATCAAGGCTGAACTAGATAAGGTCCAACCTGATGAGCAGATGac                                                                                                                                                                                                      <  1:266026/301‑1 (MQ=255)`  
                                                                                                       `gCGTTCAGTGACGCTTCTAGCCGTGCTGGTGGTGCTGACTTCCTGATGCGAGTCGGTGACAAGAAGGTAACACTTAACGGAGCCACTACGACTTACCGAGAGTTGATTGGTGAGGAACAGTGGAACGCTCTCATGGTCACAGCACAACGTTCTCAGTTTGAGACTGACGCGAAGCTGAACGAGCAGTATCGCTTGGAGATTAACTCTGCGCTGAACCAAGAGGACCCAAGGACAGCTTGGGAGATGCTTCAAGGTATCAAGGCTGAACTAGATAAGGTCCAACCTGATGAGCAGATGacac                                                                                                                                                                                                    >  1:427453/1‑301 (MQ=255)`  
                                                                                                        `cGTTCAGTGACGCTTCTAGACGTGCTGGTGGTGCTGACTTCCTGATGCGAGTCGGTGACAAGAAGGTAACACTTAACGGAGCCACTACGACTTACCGAGAGTTGATTGGTGAGGAACAGTGGAACGCTCTCATGGTCACAGCACAACGTTCTCAGTTTGAGACTGACGCGAAGCTGAACGAGCAGTATCGCTTGGAGATTAACTCTGCGCTGAACCAAGAGGACACAAGGACAGCGTGGGAGATCATTCAATGTATCAAGGCTGAACTAGATAAGGTCCAACCTGATGAGCAAATGAgacc                                                                                                                                                                                                   >  1:209960/1‑301 (MQ=255)`  
                                                                                                          `gagatgtgtataagataaagTGCTGGTGGTGCTTACTTCCTGAGGCGAGGCGGTTACAAGAAGGTAGCGCTTAACGGAGCCAATACGACTTACCGAGAGTTGATTGGTGAGTAACAGTGGAAAGCTCTCATGGTCACAGCACAACGTTCTCAGTTTGAGACTGATGCGAAGCTGAACGAGCAGTATCGCTTGGAGATTAACTCTGCGCTGAACCAAGAGGACCCAAGGACAGCTTGGGAGATGCTTCAAGGTATCAAGGCTGAACTAGATAAGGTCCAACCTGATGAGCAGATGAcaccac                                                                                                                                                                                                 <  1:80685/282‑1 (MQ=255)`  
                                                                                                            `ggacagcGCTTCTAGCCGTGCTGGTGGTGCTGACTTCCTGATGCGAGTCGGTGACAAGAAGGTAACACTTAACGGAGCCACTACGACTTACCGAGAGTTGATTGGTGAGGAACAGTGGAACGCTCTCATGGTCACAGCACAACGTTCTCAGTTTGAGACTGACGCGAAGCTGAACGAGCAGTATAGCTTGGAGATTAACTCTGCGCTGAACCAAGAGGACCCAAGGACAGCTTGGGAGATGCTTCAAGGTATCAAGGCTGAACTAGATAAGGTCCAACCTGATGAGCAGATGACACCACaa                                                                                                                                                                                               <  1:372698/295‑1 (MQ=255)`  
                                                                                                                  `cGCTTCTAGCCGTGCTGGTGGTGCTGACTTCCTGATGCGAGTCGGTGACAAGAAGGTAACACTTAACGGAGCCACTACGACGTACCGAGAGTTGATTGGTGAGGAACAGTGGAACGCTCTCATGGTCACAGCACAACGTTCTCAGTTTGAGACTGACGCGAAGTTGAACGAGCAGTATCGCTTGGAGATTAACTCTGCGCTGAACCAAGAGGACCCAAGGACAGCTTGGGAGATGCTTCAAGGTATCAATGCTGAACTAGATAAGGTCCAACCTGATGAGCAGATGACACCACAACGTGAg                                                                                                                                                                                         <  1:239442/301‑1 (MQ=255)`  
                                                                                                                    `cTTCTAGCCGTGCTGGTGGTGCTGACTTCCTGATGCGAGTCGGTGACAAGAAGGTAACACTTAACGGAGCCACTACGACTTACCGAGAGTTGATTGGTGAGGAACAGTGGAACGCTCTCATGGTCACAGCACAACGTTCTCAGTTTGAGACTGACGCGAAGCTGAACGAGCAGTATCGCTTGGAGATTAACTCTGCGCTGAACCAAGAGGACCCAAGGACAGCTTGGGAGATGCTTCAAGGTATCAAGGCTGAACTAGATAAGGTCCAACCTGATGAGCAGATGAcaccctgtctcttata                                                                                                                                                                                       >  1:473575/1‑289 (MQ=255)`  
                                                                                                                      `tcgaGCTGTGCTGGTGGTGCTGACTTCCTGATGCGAGTCGGTGACAAGAAGGTAACACTTAACGGAGCCACTACGACTTACCGAGAGTTGATTGGTGAGGAACAGTGGAACGCTCTCATGGTCACAGCACAACGTTCTCAGTTTGAGACTGACGCGAAGCTGAACGAGCAGTATCGCTTGGAGATTAACTCTGCGCTGAACCAAGAGGACCCAAGGACAGCTTGGGAGATGCTTCAAGGTATCAAGGCTGAACTAGATAAGGTCCAACCTGATGAGCAGATGACACCACAACGTGAGTGGc                                                                                                                                                                                     <  1:176047/298‑1 (MQ=255)`  
                                                                                                                           `ccGTTCTGGTGGTGCTGACTTCCTGATGCGAGTCGGTGACAAGAAGGTAACACTTAACGGAGCCACTACGACTTACCGAGAGTTGATTGGTGAGGAACAGTGGAACGCTCTCATGGTCACAGCACAACGTTCTCAGTTTGAGACTGACGCGAAGCTGAACGAGCAGTATCGCTTGGAGATTAACTCTGCGCTGAACCAAGAGGACCCAAGGACAGCTTGGGAGATGCTTCAAGGTATCAAGGCTGAACTAGATAAGGTCCAACCTGATGAGCAGATGACACCACAACGTGAGTGGCTAAtc                                                                                                                                                                                <  1:440300/301‑1 (MQ=255)`  
                                                                                                                                  `ggtggtGCTGACTTCCTGATGCGAGTCGGTGACAAGAATGTAACACTTAACGGAGCCACTACGACTTACCGAGAGTTGATTGGTGAGGAACAGTGGAACGCTCTCATGGTCACAGCACAACGTTCTCAGTTTGAGACTGACGCGAAGCTGAACGAGCAGTATCGCTTGGAGATTAACTCTGCGCTGAACCAAGAGGACCCAAGGACAGCTTGGGAGATGCTTCAAGGTATCAAGGCTGAACTAGATAAGGTCCAACCTGATGAGCAGATGACACCACAACGTGAGTGGCTAATCTCCGcac                                                                                                                                                                         <  1:26025/301‑1 (MQ=255)`  
                                                                                                                                  `ggtggtGCTGACTTCCTGATGCGAGTCGGTGACAAGAAGGTAACACTTAACGGAGTCACTACGACTTACCGAGAGTTGATTGGTGAGGAACAGTGGAACGCTCTCATGGTCACAGCACAACGTTCTCAGTTTGAGACTGACGCGAAGCTGAACGAGCAGTATCGCTTGGAGATTAACTCTGCGCTGAACCAAGAGGACCCAAGGACAGCTTGGGAGATGCTTCAAGGTATCAAGGCTGAACTAGATAAGGTCCAACCTGATGAGCAGATGACACCACAACGTGAgctgtctcttatacaca                                                                                                                                                                         >  1:507515/1‑285 (MQ=255)`  
                                                                                                                                   `gtggtgCTGACTTCCTGATGCGAGTCGGTGACAAGAAGGTAACACTTAACGGAGCCACTACGACTTACCGAGAGTTGATTGGTGAGGAACAGTGGAACGCTCTCATGGTCACAGCACAACGTTCTCAGTTTGAGACTGACGCGAAGCTGAACGAGCAGTATCGCTTGGAGATTAACTCTGCGCTGAACCAAGAGGACCCAAGGACAGCTTGGGAGATGCTTCAAGGTATCAAGGCTGAACTAGATAAGGTCCAACCTGATGAGCAGATGACACCACAACGTGAGTGGCTAATCTCCGcaca                                                                                                                                                                        >  1:107728/1‑301 (MQ=255)`  
                                                                                                                                    `gggtgCTGACTTCCTGATGCGAGTCGGTGACAAGAAGGTAACACTTAACGGAGCCACTACGACTTACCGATAGTTGATTGGTGAGGAACAGTGGAACGCTCTCATGGTCACAGCACAACGGTCTCAGTTTGAGACTGACGCGAAGCTGAACGAGCAGTATCGCTTGGAGATTAACTCTGCGCTGAACCAAGAGGACCCAAGGACAGCTTGGGAGATGCTTCAAGGTATCAAGGCTGAACTAGATAAGGTCCAACCTGATGAGCAGATGACACCACAACGTGAGTGGCTAATCTCCGCACAg                                                                                                                                                                       <  1:156069/300‑1 (MQ=255)`  
                                                                                                                                        `gCTGACTTCCTGATGCGAGTCGGTGACAAGAAGGTAACACTTAACGGAGCCACTACGACTTACCGAGAGTTGATTGGTGAGGAACAGTGGAACGCTCTCATGGTCACAGCACAACGTTCTCAGTTTGAGACTGACGCGAAGCTGAACGAGCAGTATCGCTTGGAGATTAACTCTGCGCTGAACCAAGAGGACCCAAGGACAGCTTGGGAGATGCTTCAAGGTATCAAGGCTGAACTAGATAAGGTCCAACCTGATGAGCAGATGACACCACAACGTGAGTGGCTAATCTCCGCACAGGaaa                                                                                                                                                                   >  1:206009/1‑300 (MQ=255)`  
                                                                                                                                             `cTTCCTGATGCGAGTCGGTGACAAGAAGGTAACACTTAACGGAGCCACTACGACTTACCGAGAGTTGATTGGTGAGGAACAGTGGAACGCTCTCATGGTCACAGCACAACGTTCTCAGTTTGAGACTGACGCGAAGCTGAACGAGCAGTATCGCTTGGCGATTAACTCTGCGCTGAAACAAGAGGACACAAGGACAGATTGGGAGATGCTGCAAGGTATCAAGGCTGAACTAGATAAGGTCCAACCTGATGAGCAGATGACACCACCACGTGAGTGGCTaagcctgtctcttatacacatc                                                                                                                                                              >  1:62050/1‑281 (MQ=255)`  
                                                                                                                                                   `gATGGGAGTCGGTGACAAGAAGGTAACACTTAACGGAGCAACTACGACTTATCGAGAGTTGATTGGTGAGGAACAGTGGAACGCTCTCATGGTCACAGCACAACGTTATCAGTGTGAGACTGACGCGAAGCTGAACGAGCAGTATCGCTTGGAGAGTAACTCTGCGCTGAACCAAGAGGACCCAAGGACAGCTTGGGAGATGCTTCAAGGTATCAAGGCTGAACTAGATAAGGTCCAACCTGATGAGCAGATGACACCACAACGTGAGTGGCTAATCTCCGCACAGGAACAAGTTCAGAAt                                                                                                                                                        <  1:124491/301‑1 (MQ=255)`  
                                                                                                                                                    `aTGCGAGGCGGTGACAAGAAGGTAACACTTAACGGAGGCACTACGACTTACCGAGAGTTGATTGGTGAGGAACCGTGGAACGCTCTCATGTTCACAGCACAACGTTCTCAGTTTGAGACTGACGCGAAGCTGAACGAGCAGTATCGCTTGGAGATTAACTCTGCGATGAACCAAGAGGACCCAAGGACAGCTTGGGAGATGCTTCAAGGTATCAAGGCTGAACTAGATAAGGTCCAACCTGATGAGCAGATGACACCACAACGTGAGTGGCTAATCTCCGCACAGGAACAAGTTCAGAATc                                                                                                                                                       <  1:256179/301‑1 (MQ=255)`  
                                                                                                                                                         `cggagatggggataaagggtagcTTAACGGAGCCACTACGACTTACCGAGAGTTGATTGGTGAGGAACAGTGGAACGCTCTCATGGTCACAGCACAACGTTCTCAGTTTGAGACTGACGCGAAGCTGAACGAGCAGTATCGCTTGGAGATTAACTCTGCGCTGAACCAAGAGGACCCAAGGACAGCTTGGGAGATGCTTCAAGGTATCAAGGCTGAACTAGATAAGGTCCAACCTGATGAGCAGATGACACCACAACGTGAGTGGCTAATCTCCGCACAGGAACAAGTTCAGAATCAGATg                                                                                                                                                  <  1:382434/279‑1 (MQ=255)`  
                                                                                                                                                                     `gaagGTAACACTTAACGGAGCCACTACGACTTACCGAGAGTTGATTGGTGAGGAACAGTGGAACGCTCTCATGGTCACAGCACAACGTTCTCAGTTTGAGACTGACGCGAAGCTGAACGAGCAGTATCGCTTGGAGATTAACTCTGCGCTGAACCAAGAGGACCCAAGGACAGCTTGGGAGATGCTTCAAGGTATCAAGGCTGAACTAGATAAGGTCCAACCTGATGATCAGATGACACCACAACGTGAGTGGCTAATCTCCGCACAGGAACAAGTTCAGAATCAGATTAACGCATGGACg                                                                                                                                      >  1:361388/1‑301 (MQ=255)`  
                                                                                                                                                                            `acgctcaaCGGAGCCACAACGCATTACCGAGAGTTGATTGGTGAGGAACAGTGGAACGCTCACATTGTCACAGCACAACGTTCTCAGTTTGAGACTGACGCGAAGCTGAACGAGCAGTATCGCTTGGAGATTAACTCTGCGCTGATCCAAGAGGACCCAAGGACAGCTTGGGAGATGCTTCAAGGTATCAAGGCTGAACTCGATAAGGTCCAACCTGATGAGCAGATGACACCACAACGTGAGTGGCTAATCTCCGCACAGGAACAAGTTCAGAATCAGATGAACGCATGGACGAAAGCTc                                                                                                                               <  1:298782/295‑1 (MQ=255)`  
                                                                                                                                                                            `acacTTAACGGAGCCACTACGACTTACCGAGAGTTGATTGGTGAGGAACAGTGGAACGCTCTCATGGTCACAGCACAACGTTCTCAGTTTGAGACTGACGCGAAGCTGAACGAGCAGTATCGCTTGGAGATTAACTCTGCGCTGAACCAAGAGGACCCAAGGACAGCTTGGGAGATGCTTCAAGGTATCAAGGCTGAACTAGATAAGGTCCAACCTGATGAGCAGATGACACCACAACGTGAGTGGCTAATCTCCGCACAGGAACAAGTTCAGAATCAGATGAACGCAGGGACGAAAGCTc                                                                                                                               >  1:252937/1‑301 (MQ=255)`  
                                                                                                                                                                             `cacTTAACGGAGCCACTACGACTTACCGAGAGTTGATTGGTGAGGAACAGTGGAACGCTCTCATGGTCACAGCACAACGTTCTCAGTTTGAGACTGACGCGAAGCTGAACGAGCAGTATCGCTTGGAGATTAACTCTGCGCTGAACCAAGAGGACCCAAGGACAGCTTGGGAGATGCTTCAAGGTATCAAGGCTGAACTAGATAAGGTCCAACCTGATGAGCAGATGACACCACAACGTGAGTGGCTAATCTCCGCACAGGAACAAGTTCAGAATCAGATGAACGCATGGACGAAAGCTCa                                                                                                                              >  1:304146/1‑301 (MQ=255)`  
                                                                                                                                                                             `cacTTAACGGAGCCACTACGACTTACCGAGAGTTGATTGGTGAGGAACAGTGGAACGCTCTCATGGTCACAGCACAACGTTCTCAGTTTGAGACTGACGCGAAGCTGAACGAGCAGTATCGCTTGGAGATTAACTCTGCGCTGAACCAAGAGGACCCAAGGACAGCTTGGGAGATGCTTCAAGGTATCAAGGCTGAACTAGATAAGGTCCAACCTGATGAGCAGATGACAACACAACGTGAGTGGCTAATCTCCGCACAGGAACAAGTTCAGAATCAGATGAACGCCTGGACGAAAGcccc                                                                                                                              >  1:289279/1‑298 (MQ=255)`  
                                                                                                                                                                               `cTTAATGGAGCCACTACGACTTACCGAGAGTTGATTGGTGAGGAACAGTGGAACGCTCTCATGGTCACAGCACAACGTTCTCAGTTTGAGACTGACGCGAAGCTGAACGAGCAGTATCGCTTGGAGATTAACTCTGCGCTGAACCAAGAGGACCCAAGGACAGCTTGGGAGATGCTTCAAGGTATCAAGGCTGAACTAGATAAGGTCCAACCTGATGAGCAGATGACACCACAACGTGAGTGGCTAATCTCCGCACAGGAACAAGTTCAGAATCAGATGAACACATGGActgtctcttata                                                                                                                            >  1:85214/1‑290 (MQ=255)`  
                                                                                                                                                                                      `ctggcACTACGACTTACCGAGAGTTGAGTGTGAAGGAACAGTGGAACGCTATCATGGGCACATCACAACGTTCTCAGTTTGAGACGGACGTGAAGCTGAACGAGCAGTATCGCTTGGAGATTAACTCTGCGCTGAACCAAGATGACCCAAGGACAGCTTGGGAGATGCTTCAAGGTATCAAGGCTGAACTAGATAAGGTCCAACCTGATGAGCAGATGACACCACAACGTGAGTGGCTAATCTCCGCACAGGAACAAGTTCAGAATCAGATGAACGCATGGACGAAAGCTCAGGCCAAGGc                                                                                                                     <  1:315674/297‑1 (MQ=255)`  
                                                                                                                                                                                        `gCCACTACGACTTACCGAGAGTTGATTGGTGAGGAACAGTGGAACGCTCTCATGGTCACAGCACAACGTTCTCAGTTTGAGACTGACGCGAAGCTGAACGAGCAGTATCGCTTGGAGATTAACTCTGCGCTGAACCAAGAGGACCCAAGGACAGCTTGGGAGATGCTTCAAGGTATCAAGGCTGAACTAGATAAGGTCCAACCTGATGAGCAGATGACACCACAACGTGAGTGGCTAATCTCCGCACAGGAACAAGTTCAGAATCAGATGAACGCATGGACGAAAGCTCAGGCCAAGGctc                                                                                                                   >  1:49862/1‑301 (MQ=255)`  
                                                                                                                                                                                          `cACTACGACTTACCGAGAGTTGATTGGTGAGGAACAGTGGAACGCTCTCATGGTCACAGCACAACGTTCTCAGTTTGAGACTGACGCGAAGCTGAACGAGCAGTATCGCTTGGGGATTAACTCTGCGCTGAACCAAGAGGACCCAAGGACAGCTTGGGAGATGCTTCAAGGTATCAAGGCTGAACTAGATAAGGTCCAACCTGATGAGCAGATGACACCACAACGTGAGTGGCTAATCTCCGCACAGGAACAAGGTCAGAATCAGATGAACGCATGGACGCAAGCTCAGGCCAAGGCTATg                                                                                                                 >  1:449652/1‑301 (MQ=255)`  
                                                                                                                                                                                          `cACTACGACTTACCGAGAGTTGATTGGTGAGGAACAGTGGAACGCTCTCATGGTCACAGCACAACGTTCTCAGTTTGAGACTGACGCGAAGCTGAACGAGCAGTATCGCTTGGAGATTAACTCTGCGCTGAACCAAGAGGACCCAAGGACAGCTTGGGAGAGTATTCAAGGTATCAAGGCTGAACTAGATAAGGTCCAACCTGATGAGCAGATGACACCACAACGTGATTGGCTAATCTCCGAACCGGAACACGTTCAGAAACAGAGGAACGAATGGAGGAAAGCTCAGGCCAAGGCTCTg                                                                                                                 >  1:278778/1‑301 (MQ=255)`  
                                                                                                                                                                                            `agaagACTAAACGAGAGTTGATTGGTGAGGAACAGTCGAACGCTCTCATGGTCAAAGCACAACGTTCTCACTTTGAGACTGACGAGAAGCTGAACGAGCAGTATCGCTTGGATATGAACTCTGCGCTTAACCAAGAGGACCCAAGGACAGCTTGGGAGATGCTTCAAGGTATCAAGGCTGAACTAGATAAGGTCCAACATGATGAGGATATGACACCACAACGTGAGTGTCTAATCTCCGCACAGGAAAAAGTTCAG‑ATCAGATGAACGCATGGACGAAAGCTCAGGTCAAGGCTCTGGAc                                                                                                              <  1:112216/297‑1 (MQ=255)`  
                                                                                                                                                                                             `tACGACTTACCGAGAGTTGATTGGTGAGGAACAGTGGAACGCTCTCATGGTCACAGCACAACGTTCTCAGTTTGAGACTGACGCGAAGCTGAACGAGCAGTATCGCTTTGAGATTAACTCTGCGCTGAACCAAGAGGACCCAAGGACAGCTTGGGAGATGCTTTAAGGTATCAAGGCTGAACTAGATAAGGTCCAACCTGATGAGCAGATGACACCACAACGAGAGTGGCTAATCTCCGCACAGGAACAAGTTCAGAATCAGATGAACGCATGGACGAAAGCTCAGGCCAAGGCTCTGGAc                                                                                                              <  1:235866/301‑1 (MQ=255)`  
                                                                                                                                                                                                  `agagacaggagTTGATTGGTGAGGAACAGTGGAACGCTCTCATGGTCACAGCACAACGTTCTCAGTTTGAGACTGACGCGAAGCTGAACGAGCAGTATCGCTTGGAGATTAACTCTGCGCTGAACCAAGAGGACCCAAGGACAGCTTGGGAGATGCTTCAAGGTATCAAGGCTGAACTAGATAAGGTCCAACCTGATGAGCAGATGACACCACAACGTGAGTGGCTAATCTCCGCACAGGAACAAGTTCAGAATCAGATGAACGCATGGACGAAAGCTCAGGCCAAGGCTCTGGACGATTc                                                                                                         <  1:270393/293‑1 (MQ=255)`  
                                                                                                                                                                                                     `aCCGAGAGTTGATTGGTGAGGAACAGTGGAACGCTCTCATGGTCACAGCACAACGTTCTCAGTTTGAGACTGACGCGAAGCTGAACGAGCAGTATCGCTTGGAGATTAACTCTGCGCTGAACCAAGAGGACCCAAGGCCAGCTTGGGAGAAGCTTCAAGGTATCACGGCTGAACTAGATAAGGTCCCACCTGATGAGCAGATGAAACCACAACGTAAGTGACTTATCTCGGCTACGGAACAAGTTCAGAATCCGATGAACGAATGGCACAAGGCCCAGGccctggcgcttatcaacatatc                                                                                                      >  1:242919/1‑281 (MQ=255)`  
                                                                                                                                                                                                                    `gTGAGGAACAGTGGAACGCTCTCATGGTCACAGCACAACGTTCTCAGTTTGAGACTGACGCGAAGCTGAACGAGCAGTATCGCTTGGAGATTAACTCTGCGCTGAACCAAGAGGACCCAAGGACAGCTTGGGAGATGCTTCAAGGTATCAAGGCTGAACTAGATAAGGTCCAACCTGATGAGCAGATGACACCACAACGTGAGTGGCTAATCTCCGCACAGGAACAAGCTCAGAATCAGATGAACGCATGGACGAAAGCTCAGGCCAGAGCTCTGGACGTGTCCATGAAGTCAATGAACaa                                                                                       >  1:138326/1‑301 (MQ=255)`  
                                                                                                                                                                                                                        `ggAACAGTGGAACGCTCTCATGGTCACAGCACAACGTTCTCAGTTTGAGACTGACGCGAAGCTGAACGAGCAGTATCGCTTGGAGATTAACTCTGCGCTGAACCAAGAGGACCCAAGGACAGCTTGGGAGATGCTTCAAGGTATCAAGGCTGAACTAGATAAGGTCCAACCTGATGAGCAGATGACACCACAACGTGAGTTGCTAATCTCCGCACAGGAACAAGTTCAGAATCAGATGAACGCATGGACGAAAGCTCAGGCCAAGGCTCTGGACGATTCCATGAAGTCAAGGAAcctgtct                                                                                   >  1:100080/1‑295 (MQ=255)`  
                                                                                                                                                                                                                           `aCAGTGGAACGCTCTCATGGTCACAGCACAACGTTCTCAGTTTGAGACTGACGCGAAGCTGAACGAGCAGTATCGCTTGGAGATTAACTCTGCGCTGAACCAAGAGGACCCAAGGACAGCTTGGGAGATGCTTCAAGGTATCAAGGCTGAACTAGATAAGGTACAACCTGATGAGCAGATGACACCACAACGTGAGTGGCTAATATCCGCACAGGAACAAGTTCAGAATCAGATGAACGCATGGACGAAAGCTCAGGCCAAGGCTCTGGACGATTCCATGAAGTCAATGAACAAACTTGAc                                                                                >  1:454813/1‑301 (MQ=255)`  
                                                                                                                                                                                                                              `gTGGAACGCTCTCATGGTCACAGCACAACGTTCTCAGTTTGAGACTGACGCGAAGCTGAACGAGCAGTATCGCTTGGAGATTAACTCTGCGCTGAACCAAGAGGACCCAAGGACAGCTTGGGAGATGCTTCAAGGTATCAAGGCTGAACTAGATAAGGTCCAACCTGATGAGCAGATGACACCACAACGTGAGTGGCTAATCTCCGCACAGGAACAAGTTCAGAATCAGATGAACGCATGGACGAAAGCTCAGGCCAAGGCTCTGGACGATTCCATGAAGTCAATGAACAAACTTGACGTa                                                                             >  1:18028/1‑301 (MQ=255)`  
                                                                                                                                                                                                                                `ggAACGCGCTCATGGTCAAAGAACAACGTTCTCAGTTTGAGACTGACGCGAAGCTGAACGAGCAGTATCGCTTGGAGATTAACTCTGCGCTGAACCAAGAGGACCCAAGGACAGCTTGGGAGATGCTTCAAGGTATCAAGGCTGAACTAGATAAGGTCCAACCTGATGAGCAGATGACACCACAACGTGAGTGGCTAATCTCCGCACAGGAACAAGTTCAGAATCAGATGAACGCATGGACGAAAGCTCAGGCCAAGGCTCTGGACGATTCCATGAAGTCAATGAACAAACTTGACGTAAt                                                                           <  1:319278/301‑1 (MQ=255)`  
                                                                                                                                                                                                                                  `aaCACTCTCATGGTCACAGCACAACGTTCTCAGTTTGAGACTGACGCGAAGCTGAACGAGCAGTATCGCTTGGAGATTAACTCTGCGCTGAACCAAGAGGACCCAAGGACAGCTTGGGAGATGCTTAAAGGTATCAAGGCTGAACTAGATAAGGTCCAACCTGATGAGCAGATGACACCACAACGTGAGTGGCTAATCTACGCACAGGAACAAGTTCAGAATCAGATGAACGCATGGACGAAAGCTAAGGCTAAGGCTCTGGACGATTCCATGAAGTCAATGAACAAACTTGACGTAATCg                                                                         <  1:420349/301‑1 (MQ=255)`  
                                                                                                                                                                                                                                    `cGCTCTCATGGTCACAGCACAACGTTCTCAGTTTGAGACTGACGCGAAGCTGAACGAGCAGTATCGCTTGGAGATTAACTCTGCGCTGAACCAAGAGGACCCAAGGACAGCTTGGGAGATGCTTCAAGGTATCAAGGCTTAACTAGATAAGGTCCAACCTGATGAGCAGATGACACCACAACGTGAGTGGCTAATCTCCGCACAGGAACAAGTTCAGAATCAGATGAACGCATGGACGAAAGCTCAGGCCAAGGCTCCGGACGATTCCATGAAGTCAATGAACAAACTTTACGTAATCgta                                                                       >  1:468105/1‑299 (MQ=255)`  
                                                                                                                                                                                                                                          `cATGGTCACAGCACAACGTTCTCAGTTTGAGACTTACGCGAAGCTGAACGAGCAGTATCGCTTGGAGATTAACTCTGCGCTGAACCAAGAGGACCCAAGGACAGCTTGGGAGATGCTTCAAGGTATCAAGGCTGAACTAGATAAGGTCCAACCTGATGAGCAGATGACACCACAACGTGAGTGGCTAATCTCCGCACAGGAACAAGTTCAGAATCAGATGAACGCATGGACGAAAGCTCAGGCCAAGGCTCTGGACGAATCCATGAAGTCAATGAACAAACTTGACGTAATCGACAAGCaa                                                                 >  1:343157/1‑301 (MQ=255)`  
                                                                                                                                                                                                                                              `gTCACAGCACAACGTTCTCAGTTTGAGACTGACGCGAAGCTGAACGAGCAGTATCGCTTGGAGATTAACTCTGCGCTGAACCAAGAGGACCCAAGGACAGCTTGGGAGATGCTTCAAGGTATCAAGGATGAACTAGATAAGGTCCAACCTGATGAGCAGATGACACCACAACGTGAGTGGCTAATCTCCGCACAGGAACAAGTTCAGAATCAGATGAACGCATGGACGAAAGCTCAGGCCAAGGCTCTGGACGATTCCATGAAGTCAATGAACAAACTTGACGTAATCGACAAGCAATTcc                                                             >  1:337527/1‑301 (MQ=255)`  
                                                                                                                                                                                                                                                  `caGCACAACGTTCTCAGTTTGAGACTGACGCGAAGCTGAACGAGCAGTATCGCTTGGAGATTAACTCTGAGCTGAACAAAGAGGACCCAAGGCCAGCTTGGGAGATGCTTCAATGTATCAAGGCTGAACTAGATCAGGTCCAACCTGATGAGCAGATTACACCACAACGTGTGTTGCTAATCTCCGCACAGGAACACGTTCAGAATCAGATGAACGCATGGACGAAAGCTTAGGCCCCGGCTCTGGACGATTCCATGAAGTCAATGAACAACCTTGACGTCATAGACCACCAATTACAGaa                                                         >  1:20322/1‑301 (MQ=255)`  
                                                                                                                                                                                                                                                   `aacacaACGTTCTAAGTTTGAGAATGACGCGAAGCTGAACGAGCAGTATCGCTTGGAGATTAACTCTTCGATGAACCAAGAGGACCCAAGGACAGCTTGGGAGATGCTTCAAGGTATCAAGGCTGAACTAGATAAGGTCCAACCTGATGAGCAGATGACACCACAACGTGAGTGGCTAATCTCCGCACAGGAACAAGTTCAGAATCAGATGAACGCATGGACGAAAGCTCAGGCCTAGGCTCTGGACGATTCCATGAAGTCAATGAACAAACTTGACGTAATCGACAAGCAATTCCAGAAg                                                        <  1:388196/299‑1 (MQ=255)`  
                                                                                                                                                                                                                                                    `gCATAACGTTCTCAGTTTGAGACTGACGCGAAGCTGAACGAGCAGTATCGCTTGGAGATTAACTCTGCGCTGAACCAAGAGGACCCAAGGACAGCTTGGGAGATGCTTCAAGGTATCAAGGCTGAACTAGATAAGGTCCAACCTGATGAGCAGATGACACCACAACGTGAGTGGCTAATCTCCGCACAGGAACAAGTTCAGAATCAGATGAACGCATGGACGAAAGCTCAGGCCAAGGCTCTGGACGATTCCATGAAGTCAATGAACAAACTTGACGTAATCGACAAGCAATTCCAGAAGc                                                       >  1:436908/1‑301 (MQ=255)`  
                                                                                                                                                                                                                                                         `aCGTTCTCAGTTTGAGACTGACGCGAAGCTGAACGAGCTGTATCGCTTGGAGATTAACTCTGCGCTGAACCAAGAGGACCCAAGGACAGCTTGGGAGATGCTTCACGGTATTAAGGCTGAACTAGATAAGGTCCAACCTGATGAGCAGATGACACCACAACGTGAGTGGCTAATCTCCGCACAGGACCAAGTTCAGAATCAGATGAACGCATGGACGAAAGCTCAGGCCAAGGCTCTGGACGATTCAATGAAGTCAATGAACAAACTTGACGTAATCGACAAGCAATTCCAGAAGCGAATc                                                  >  1:293736/1‑301 (MQ=255)`  
                                                                                                                                                                                                                                                         `aCGTTCTCAGTTTGAGACTGACGCGAAGCTGAACGAGCAGTATCGCTTGGAGATTAACTCTGCGCTGAACCAAGAGGACCCAAGGACAGCTTGGGAG‑TGCTTCAAGGTATCAAGGCTGAACTAGATAAGGTCCAACCTGATGAGCAGATGACACCACAACGTGAGTGGCTAATCTCCGCACAGGAACAAGTTCAGCATCAGATGAACGCATGGACGAAAGCTCAGGCCAAGGCTCTGGACGATTCCATGAAGTCAATGAACAAACTTGCCGTAATCGACAAGCAATTCCAGAAGCGAATcc                                                 >  1:197944/1‑300 (MQ=255)`  
                                                                                                                                                                                                                                                                `cAGTTTGAGACTGACGCGAAGCTAAACGAGCAGTATCGCTTGGAGATTAACTCTGCGCTGAACCAAGAGGACCCAAGGACAGCTTGGGAGATGCTTCAAGGTATCAAGGCTGAACTAGATAAGGTCCAACCTGATGAGCAGATGACACCACAACGTGAGTGGCTAATCTCCGCACAAGAACAAGTTCAGAATCAGATGAACGCATGGACGAAAGCTCAGGCCAAGGCTCTGGACGATTCCATGAAGTCAATGAACAAACTTGACGTAATCGACAATCAATTCCAGAAGCGAATCAACTGtt                                           >  1:489299/1‑300 (MQ=255)`  
                                                                                                                                                                                                                                                                  `gTTTGAGACTGACGCGAAGCTGAACGAGCAGTATCGCTTGGAGATTAACTCTGCGCTGAACCAAGAGGACCCAAGGACAGCTTGGGAGATGCTTCAAGGTATCAAGGCTGAACTAGATAAGGTCCAACCTGATGAGCAGATGACACCACAACGTGAGTGGCTAATCTCCGCACAGGAACAAGTTCAGAATCAGATGAACGCATGGACGAAAGCTCAGGCCAAGGCTCTGGACGATTCCATGAAGTCAATGAACAAACTTGACGTAATCGACAAGCAATTCCAGAAGCGAATCAACGGTTAg                                         >  1:485898/1‑301 (MQ=255)`  
                                                                                                                                                                                                                                                                      `ggctcggagatgtgtataagagacaggTATCGCTTGGAGATTAACTCTGCGCTGAACCAAGAGGACCCAAGGACAGCTTGGGAGATGCTTCAAGGTATCAAGGCTGAACTAGATAAGGTCCAACCTGATGAGCAGATGACACCACAACGTGAGTGGCTAATCTCCGCACAGGAACAAGTTCAGAATAAGATGAACGCATGGACGAAAGCTCAGGCCAAGGCTCTGGACGATTCCATGAAGTCAATGAACAAACTTGACGTAATCGACAAGCAATTCCAGAAGCGAATCAACGGTGAGTggg                                     <  1:482339/275‑1 (MQ=255)`  
                                                                                                                                                                                                                                                                             `aagagAAGCTGAAATAGCAGTATCGAGTGGAGATTAACACTGAGCATAAACAAGAGGACCCAAGGACAGCTTGGTGGATGCTTCAAGGAATCAAGGATGAAATAGATAAGGTCCAACCTGAGGAGCAGATGACACCACAACGTGAGTGACTAATCTCCGCACAGGAACAAGTTCAGAATCAGATGAACGCATGGACGAAAGCTCAGGCCAAGGCTCTGGACGATTCCATGAAGTCAATGAACAAACTTGACGTAATCGACAAGCAATTCCAGAAGCGAATCAACGGTGAGTGGGTCTCAAc                              <  1:230546/297‑1 (MQ=255)`  
                                                                                                                                                                                                                                                                                    `gCTGAACGAGCAGTATCGCTTGGAGATTAACTCTGCGCTGAACCAAGAGGACCCAAGGACAGCTTGGGAGATGCTTCAAGGTATCAAGGCTGAACTAGATAAGGTCCAACCTGATTAGCAGATGACACCACAACGTGAGTGGCTAATCTCCGCACAGGAACAAGTTCAGAATCAGATGAACGCATGGACGAAAGCTCAGGCCAAGGCTCTGGACGATTCCATGAAGTCAATGAACAAACTTGACGTAATCGACAAGCAATTCCAGAAGCGAATCAACGGTGAGTGGGTCTCAACGGAtttt                       >  1:392352/1‑301 (MQ=255)`  
                                                                                                                                                                                                                                                                                       `gAACGAGCAGTATCGCTTGGAGATTAACTCTGCGCTGAACCAAGAGGACCCAAGGACAGCTTGGGAGATGCTTCAAGGTATCAAGGCTGAACTAGATAAGGTCCAACCTGATGAGCAGATGACACCACAACGTGAGTGGCTAATCTCCGCACAGGAACAAGTTCAGAATCAGATGAACGCATGGACGAAAGCTCAGGCCAAGGCTCTGGACGATTCCATGAAGTCAATGAACAAACTTGACGTAATCGACAAGCAATTCCAGAAGCGAAACAACGGTGAGTGGGTCTCAACGGATTCTAAg                    >  1:365364/1‑301 (MQ=255)`  
                                                                                                                                                                                                                                                                                           `gAGCAGTATCGCTTGGAGATTAACTCTGCGCTGAACCAAGAGGACCCAAGGACAGCTTGGGAGATGCTTCAAGGTATCAAGGCTGAACTAGATAAGGTCCAACCTGATGAGCAGATGACACCACAATGTGAGTGGCTAATCTCCGCACAGTAACAAGTTCAGAATCAGATGAACGCATGGACGAAAGCTCAGGCCAAGGCTCTGGACGCTTCCATGAAGTCAATGAACAAACTTGACGTAATCGACAAGCAATTCCAGAAGCGAATCAACGGTGAGTGGGTCTCAAAGGATTTTAAGGcta                >  1:4779/1‑301 (MQ=255)`  
                                                                                                                                                                                                                                                                                              `cAGTATCGCTTGGCGATTAACTCTGCGCTGAACCAAGAGGACCCAAGGACAGCTTGGGAGATGCTTCAAGGTATCAAGGCTGAACTAGATAAGGTCCAACCTGATGAGCAGATGACACCACAACGTGAGTGGCTAATCTCCGCACAGGAACAAGTTCAGAATCAGATGAACGCATGGACGAAAGCTCAGGCCAAGGCTCTGGACGATTCCATGAAGTCAATTAACAAACTTGACGTAATCGACAAGCAATTCCAGAAGCGAATCAACGGTGAGTGGGTCTCAACGGATTTTAAGGCTATGc             >  1:347071/1‑301 (MQ=255)`  
                                                                                                                                                                                                                                                                                                `gTATCGCTTGGAGATTAACTCTGCGCTGAACCAAGAGGACCCAAGGACAGCTTGGGAGATGCTTCAAGGTATCAAGGCTGAACTAGATAAGGTCCAACCTGATGAGCAGATGACACCACAACGTGAGTGGCTAATCTCCGCACAGGAACAAGTTCAGAATCAGATGAACGCATGGACGAAAGCTCAGGCCAAGGCTCTGGACGATTCCATGAAGTCAATGAACAAACTTGACGTAATCGACAAGCATTTCCAGAAGCGAATCAACGGTGAGTTGGTCTCAACGGATTTTAAGGATATGCCa           >  1:88531/1‑301 (MQ=255)`  

                                                                                                                                                                                                                                                                                                          `|`                                                                                                                                                                                                                                                                                                             
`GCGTCGTCCAGACTCTGCTGACTTCTTTGAGAAGTATATCGACAACGGTCTGGTTACTGGCGCAATCCCATCTGATGCTCAAGCCACACAGCTTATAAGCCAAGCGTTCAGTGACGCTTCTAGCCGTGCTGGTGGTGCTGACTTCCTGATGCGAGTCGGTGACAAGAAGGTAACACTTAACGGAGCCACTACGACTTACCGAGAGTTGATTGGTGAGGAACAGTGGAACGCTCTCATGGTCACAGCACAACGTTCTCAGTTTGAGACTGACGCGAAGCTGAACGAGCAGTATCGCTTGAAGATTAACTCTGCGCTGAACCAAGAGGACCCAAGGACAGCTTGGGAGATGCTTCAAGGTATCAAGGCTGAACTAGATAAGGTCCAACCTGATGAGCAGATGACACCACAACGTGAGTGGCTAATCTCCGCACAGGAACAAGTTCAGAATCAGATGAACGCATGGACGAAAGCTCAGGCCAAGGCTCTGGACGATTCCATGAAGTCAATGAACAAACTTGACGTAATCGACAAGCAATTCCAGAAGCGAATCAACGGTGAGTGGGTCTCAACGGATTTTAAGGATATGCCAGTCAACGAG  >  NC_001604.1/28960‑29557`

  

Alignment Legend

Aligned base mismatch/match (shaded by quality score): `ATCG/ATCG` `< 3 ≤` `ATCG/ATCG` `< 10 ≤` `ATCG/ATCG` `< 20 ≤` `ATCG/ATCG` `< 34 ≤` `ATCG/ATCG` `< 38 ≤` `ATCG/ATCG`

Unaligned base: `atcg`    Masked matching base: `atcg`    Alignment gap: `‑`    Deleted base: `‑`
